# American Spinal Injury Association Impairment Scale Grade E Conversion After Spinal Cord Injury: Incidence, Conversion Characteristics, and Impact of Age on Functional Independence

**DOI:** 10.46292/sci25-00009

**Published:** 2025-08-22

**Authors:** Gianmarco Brancato, Christian Schuld, Laura Heutehaus, Patrick Jersch, Doris Maier, Patrick Freund, Martin H. Pouw, Giorgio Scivoletto, EMSCI Study Group, Norbert Weidner, Ruediger Rupp, European Multicenter Study about Spinal Cord Injury (EMSCI), Axel Hempfing, Hans Fuerstenberg, Kerstin Rehahn, Yorck-Bernhard Kalke, Jesus Benito, Jiri Kriz, Josina Waldmann, Bjoern Zoerner, Mirko Aach, Andreas Badke, Steffen Franz, Margret Hundt-Georgiadis, Naveen Kumar, Antonio Nardone

**Affiliations:** 1 Spinal Cord Injury Center, Heidelberg University Hospital, Heidelberg, Germany; 2 Medical Faculty Heidelberg, Heidelberg University, Heidelberg, Germany; 3 Berufsgenossenschaftliche Unfallklinik Murnau, Murnau, Germany; 4 Spinal Cord Injury Center, Balgrist University Hospital, University of Zurich, Zurich, Switzerland; 5 Department of Orthopaedic Surgery, Radboud University Medical Center, Nijmegen, Netherlands; 6 Spinal Center, Spinal Rehabilitation Laboratory, IRCCS Fondazione S. Lucia, Rome, Italy; Werner-Wicker-Hospital, Bad Wildungen, Germany; SRH-Hospital, Langensteinbach, Germany; Treatment Centre for Spinal Cord Injuries, Trauma Hospital Berlin, Berlin, Germany; Spinal Cord Injury Center, Orthopaedic University Hospital Ulm, Ulm, Germany; Institut Guttmann, Barcelona, Spain; Spinal Cord Unit, Department of Rehabilitation and Sports Medicine, 2nd Faculty of Medicine, Charles University and University Hospital Motol, Prague, Czech Republic; Center for Tetra- and Paraplegia, Orthopaedic Hospital, Hessisch-Lichtenau, Germany; Swiss Paraplegic Center, Nottwil, Switzerland; Department of Spinal Cord Injuries, BG University Hospital Bergmannsheil, Bochum, Germany; BG Clinic Tuebingen, Tuebingen, Germany; Rehab Center Weisser Hof, Vienna, Austria; REHAB Basel, Basel, Switzerland; The Robert Jones and Agnes Hunt Orthopaedic Hospital, Oswestry, UK; Salvatore Maugeri Foundation, Pavia, Italy

**Keywords:** age, American Spinal Injury Association Impairment Scale E, AIS E, functional independence, ISNCSCI, SCIM, spinal cord injury

## Abstract

**Objectives:**

To investigate incidence, conversion, neurological characteristics, and age-dependent functional independence of individuals with initial spinal cord injury (SCI) recovering to American Spinal Injury Association Impairment Scale (AIS) E, meaning normal sensory and motor functions according to the International Standards for Neurological Classification of Spinal Cord Injury (ISNCSCI).

**Methods:**

We analyzed 12,221 EMSCI (European Multicenter Study about Spinal Cord Injury) ISNCSCI datasets from 5 time points over the first year after SCI of 4286 individuals (age: 48.7 ± 19 years; 23% female; 92% traumatic, 8% ischemic).

**Results:**

Sixty-five of 82 individuals with at least one AIS E exam had an initial assessment within 6 weeks after injury with neurological level of injury peaking at C4 (16.9%) and L2 (15.4%), predominantly AIS grade D (89.2%), and mean total sensory/motor scores reaching 89.4% of their maximum. First AIS E conversion was detected at a median of 171 (interquartile range 274) days after injury. A change point analysis of Spinal Cord Independence Measure (SCIM) III assessments at the time of conversion of 75 AIS E individuals demonstrates a decline of full functional independence with age particularly over 70 years (<40, 76.9%; 40-70, 42.9%; >70, 14.3%).

**Conclusion:**

The current AIS E definition insufficiently reflects the reality experienced by older people without deficits in the ISNCSCI, as functional impairments remain predominantly in mobility-related activities. To detect whether these deficits are related to comorbidities attributable to aging rather than remnant deficits of SCI, functional assessments such as the SCIM should be performed in an age-matched non-SCI control group.

## Background

Standardized classification of spinal cord injury (SCI) according to the International Standards for Neurological Classification of Spinal Cord Injury (ISNCSCI)^1^ is essential for assessment of changes in sensorimotor functions and for characterization and stratification of study participants and definition of study endpoints. It enables communication among clinicians worldwide. ISNCSCI includes a comprehensive set of classification rules with precise instructions on how to correctly determine the sensory and motor levels, the American Spinal Injury Association Impairment Scale (AIS) grade, and the zones of partial preservation. The AIS classifies the severity of SCI with 5 grades starting from sensorimotor complete (AIS A), sensory incomplete/motor complete (AIS B), sensorimotor incomplete (AIS C, D), and normal (AIS E). The AIS E grade is used in individuals with a confirmed diagnosis of SCI resulting in initial deficits but whose sensorimotor functions recover in a way that “[light touch and pinprick] sensation and motor function as tested with the ISNCSCI are graded as normal in all segments.”^1^,^2^ Although there is evidence that individuals with an initial motor incomplete lesion, particularly those graded as AIS D, have a realistic chance to recover to AIS E,^3^ little is known about the characteristics of this subpopulation.

This work aims at investigating incidence, temporal conversion pattern, and the initial neurological characteristics of individuals with acute traumatic or ischemic SCI recovering to AIS E within the first year after injury. Furthermore, we hypothesize that despite normal neurological functions according to ISNCSCI (AIS E), functional impairments still persist especially in people with higher age.

## Methods

All analyses were done in Python (version 3.12.7), using *pandas*^4^ (version 2.2.3) for data handling.

### Data preprocessing

**Figure 1.**
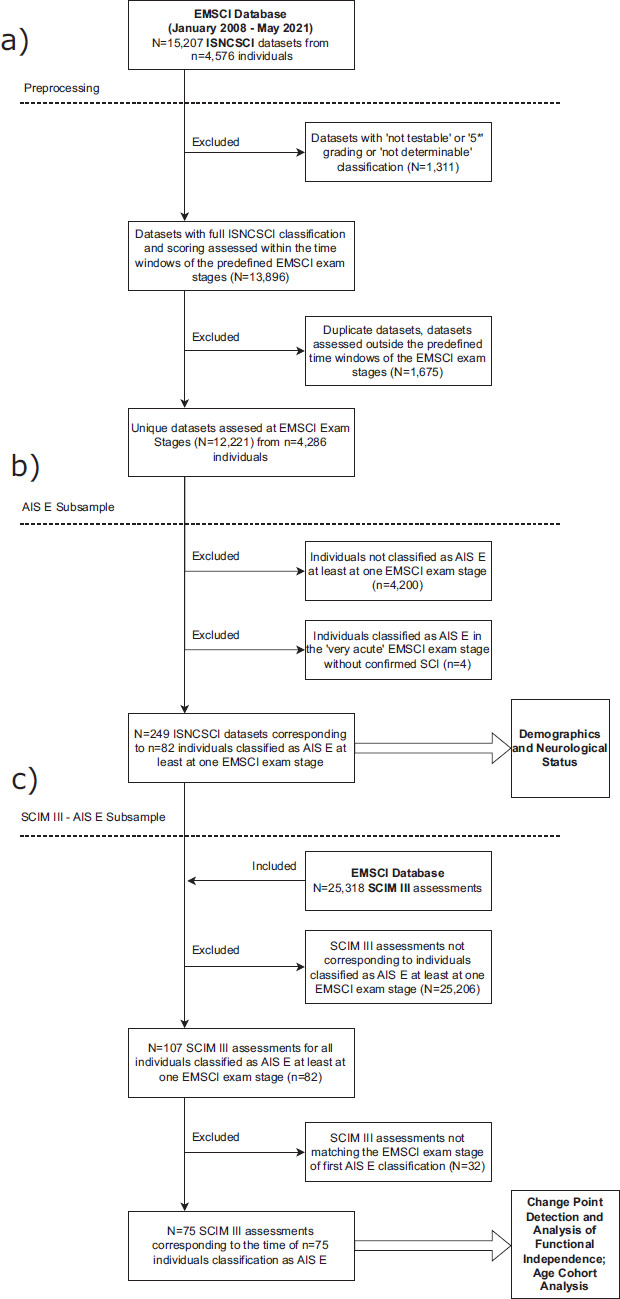
Preprocessing pipeline of the analyzed datasets. a) Initial preprocessing of the raw ISNCSCI dataset from the European Multicenter Study about Spinal Cord Injury (EMSCI) database as of May 2021. b) Further processing of the dataset for usage for the section “Demographics and Neurological Status.” c) Filtering of SCIM III assessments from the EMSCI database and combining them with the American Spinal Injury Association Impairment Scale (AIS) E dataset for analysis in the sections “Change Point Detection and Independence in Activities of Daily Living” and “Age Cohort Analysis.”

A dataset comprising 15,207 ISNCSCI assessments (January 2008 to May 2021) corresponding to 4576 individuals was sourced from the database of the European Multicenter Study about Spinal Cord Injury (EMSCI; http://emsci.org/). For more study details, see eAppendix A (clinical trials registration: NCT01571531).^5^ Initial preprocessing was performed to include only datasets with ISNCSCI examinations performed in the time windows of the predefined EMSCI exam stages, that is, very acute (0-15 days after injury [DAI]), acute I (16-40 DAI), acute II (70-98 DAI), acute III (150-186 DAI), and chronic (300-546 DAI) stage. The remaining 12,221 ISNCSCI assessments were consolidated per individual resulting in a dataset of 4286 individuals (**Figure 1a**).

To create the AIS E subsample, the dataset was filtered for individuals with at least one ISNCSCI exam stage with an AIS E grade, resulting in a dataset of 86 individuals (**Figure 1b**). From these 86 individuals, 4 individuals classified as AIS E in their first full ISNSCI exam at the very acute stage were excluded due to lack of confirmation, for example, by imaging that the initial sensorimotor deficits present after the trauma were caused by an SCI. This resulted in a dataset of 82 individuals with 249 (114 AIS E) ISNCSCI exams. For the analysis of the distribution of age and initial neurological level of injury (NLI), of these 82 individuals only those with a determinable NLI (i.e., non-AIS E classification) in one of the early (very acute or acute I) exam stages were included.

For analysis of functional impairments, 107 Spinal Cord Independence Measure (SCIM) version III^6^ assessments were available for the 82 AIS E individuals at different time points. Seventy-five assessments were available for the time point of first conversion to AIS E (**Figure 1c**) and 20 for the latest assessed time point after AIS E conversion. Only individuals with SCIM III assessments after the first AIS E conversion were included in pairwise analysis of subscale and total SCIM scores.

### Statistical analysis

Individuals were characterized in terms of conversion period, demographics, and NLI, AIS, upper/lower extremity motor scores (UEMS; LEMS), and total light touch (TLT) and total pinprick (TPP) scores. SCIM III items, subscales, and total scores were evaluated descriptively.

Significance testing was performed at a level of α ≤ 0.05. The Kruskal-Wallis one-way analysis of variance (ANOVA) implementation from *scipy*^7^ (version 1.14.1) was used to test for global significance in SCIM items between age cohorts. Two-tailed post hoc Conover-Iman testing with the implementation from *scikit-posthocs*^8^ (version 0.10.0) was performed between cohorts in SCIM items determined to have statistically significant differences. *scipy*'s implementation of a 2-sided Kendall's Tau-b correlation coefficient (τ) was used on SCIM subscales scores and total score versus age. *scipy*'s implementations of the chisquare test and 2-sided Wilcoxon signed-rank test were used respectively to test for significant differences in sex and cause (traumatic or ischemic) of SCI in subpopulations and to test for significant differences in SCIM total score between first AIS E conversion and latest available SCIM assessment.

Offline age change point detection was performed using the package *ruptures*^9^ (version 1.1.9). The change point analysis identifies the points in a data series where the mean, standard deviation, or slope of the data changes significantly. In consideration of the small size of the dataset (75 SCIM III entries; age-aligned data series was constructed from age and score) and therefore reasonable computational cost to be expected, it was decided to use an optimal algorithm. The number of assumed change points was set at 2 concordant to the number of peaks of the bimodal distribution of individuals according to age in the EMSCI database.^10^ The Dynamic Programming (DynP) model implemented in *ruptures* was used. The minimum length of segments was set to 10 to avoid segments too short to perform regression on. A least squared deviation (l2) cost function was used. Age cohort boundaries were determined via the down-rounded arithmetic mean of the change points predicted per segment in each SCIM subscale and total SCIM score.

Agreement between the cohort boundaries and the predicted age change points was evaluated using *ruptures*'s Rand Index (Δ_RI_) and Hausdorff metric (Δ_HA_) and *scikit-learn*'s^11^ (version 1.5.2) micro-averaged F score (F_mi_) implementations. Δ_RI_ is a measure of similarity between predictions and ground truth normalized to a range of 0 (total disagreement) to 1 (total agreement). Δ_HA_ denotes “the greatest temporal distance between a change point and its prediction,” where identical sets of both produce a Δ_HA_ of 0.^9^ F_mi_ is the global average of the F_1_ score, which is the harmonic mean of precision and recall, and is suitable for performance evaluation with imbalanced classes, where 0 corresponds to total disagreement and 1 to total agreement. The interquartile range (IQR) is given as the difference between the 75th and 25th percentile.

Regression for age cohorts was done using the HuberT robust linear regression implementation in *statsmodels*^12^ (version 0.14.4).

### Visualization

Plots were created using *matplotlib*^13^ (version 3.9.2) and *seaborn*^14^ (version 0.13.2).

## Results

### Demographics and neurological status

**Figure 2.**
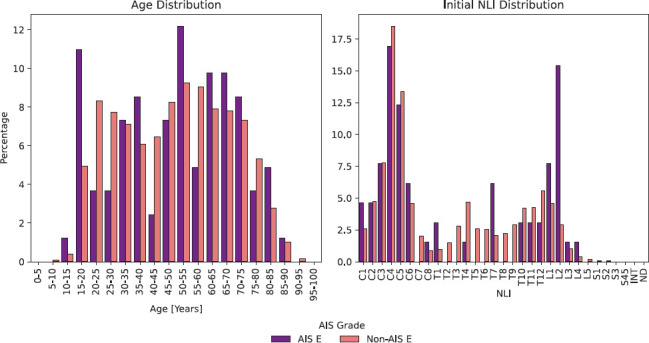
Age and neurological level of injury (NLI) distributions in the European Multicenter Study about Spinal Cord Injury (EMSCI) database (purple: American Spinal Injury Association Impairment Scale [AIS] E group = group of individuals with at least one AIS E grade in the first year after SCI); light red: non-AIS E group = group of individuals with no AIS E grade in the first year after SCI). a) Age distribution of individuals classified at least once as AIS E in the first year after SCI whose first available ISNCSCI assessment in the “very acute” (AIS E group: 51; non-AIS E group: 1919) or “acute I” (AIS E group: 14; non-AIS E group: 1802) exam stage was not AIS E, plotted as the proportion of those individuals in their respective groups. b) Distribution of the initial NLI (“very acute” or “acute I” exam stage) of the 65 individuals of the AIS E group with initial non-AIS E classification and 3721 individuals of the non-AIS E group, plotted as the proportion of those individuals in their respective groups.

The EMSCI ISNCSCI datasets contain 82 (2%) individuals with at least one exam with AIS E grade (age 49.4 ± 19.7 years; 20% female; 93.9% traumatic and 6.1% ischemic; 47.6% tetraplegia; χ^2^ *P* > .05 to whole datasets). Sixteen individuals were classified as AIS E from 2008 to 2012, 31 individuals were classified as AIS E from 2013 to 2017, and 35 individuals from 2018 to 2021 (eTable 1). Of the 82 individuals with at least one AIS E classification in the first year after SCI, 65 had an initial assessment in the early time period (i.e., very acute and/or acute stage, on average within 9.6 ± 9.3 days) after SCI with a determinable NLI (i.e., not classified as AIS E) peaking at C4 (16.9%) and L2 (15.4%) (**Figure 2**). Of those cases with at least one non-AIS E grade in the early time period after SCI, 58 were classified as AIS D (89.2%), 4 as AIS C (6.2%), and 3 as AIS B (4.6%), with the mean of the total scores (UEMS, LEMS, TLT, TPP) normalized to each maximum possible score reaching on average 89.4% (UEMS, 43.3 ± 9.8/max. 50; LEMS, 43.2 ± 11.2/max. 50; TLT, 101.9 ± 13.0/max. 112; TPP, 101.1 ± 17.0/max. 112).

First conversion to AIS E was detected at a median of 171 (IQR 274) days after injury (very acute, 3.66%, acute I, 14.63%; acute II, 23.17%; acute III, 23.17%; chronic, 35.37%). Individuals classified as AIS E already in the very acute stage were tested 11.3 ± 3.5 days post-injury. In the assessment prior to the assessment resulting in an AIS E grade (last non-AIS E grade prior to AIS E conversion: 66 [80.49%] AIS D, 2 [2.44%] AIS C, 2 [2.44%] AIS B, 12 cases [14.63%] without available prior ISNCSCI assessment), the mean of the total scores (UEMS, LEMS, TLT, TPP) normalized to each maximum possible score reached on average 92.7% (UEMS, 46.1 ± 7.3/max. 50; LEMS, 46.7 ± 7.5/max. 50; TLT, 104.5 ± 13.8/max 112; TPP, 103.1 ± 16.7/max. 112). Conversion back from AIS E to AIS D occurred in 6 individuals (7.3% of all AIS E individuals; 5 cervical, 1 thoracic) due to sensory impairments.

### Change point detection and independence in activities of daily living

SCIM III assessments were available at the exam stage of first AIS E conversion (very acute, 4.00%; acute I, 16.00%; acute II, 21.33%; acute III, 22.67%; chronic, 36.00%) for 75 individuals (46.67% tetraplegia).

Least affected SCIM III items among all individuals according to mean are respiration (100% with full independence; **Table 1**), feeding (99.56%), and grooming (98.67%). Most affected are stair management (84.89%) and mobility outdoors (83.67%). All subscale scores (self-care, respiration and sphincter management, mobility) and total SCIM score showed fair^15^ negative correlation to age (self-care: τ = −0.313, *P* ≤ .001; respiration and sphincter management: τ = −0.283, *P* ≤ .01; mobility: τ = −0.329, *P* ≤ .001; total SCIM: τ = −0.360, *P* ≤ .001).

**Table 1.** All Spinal Cord Independence Measure (SCIM) III item scores, subscale scores, and total SCIM score (marked in italics) of the exams of the 75 individuals at the exam stage of their first AIS E classification

Item	Mean score in percent, all individuals (*n* = 75)	Standard deviation in percent, all individuals	Percent cases with max. score			
			**All individuals**	**Young (<40 years old, *n* = 26)**	**Intermediate (40-70, *n* = 35)**	**Senior (>70, *n* = 14)**
*Total SCIM score*	92.17%	14.35%	49.33%	76.92%	42.86%	14.29%
Feeding	99.56%	3.85%	98.67%	100.00%	100.00%	92.86%
Bathing upper body	92.44%	20.19%	85.33%	100.00%	82.86%	64.29%
Bathing lower body	88.89%	24.71%	78.67%	100.00%	77.14%	42.86%
Dressing upper body	93.00%	19.52%	86.67%	100.00%	85.71%	64.29%
Dressing lower body	89.67%	22.92%	80.00%	96.15%	77.14%	57.14%
Grooming	98.67%	8.56%	97.33%	100.00%	100.00%	85.71%
*Self-Care subscale score*	93.47%	13.90%	72.00%	96.15%	65.71%	42.86%
Respiration	100.00%	0.00%	100.00%	100.00%	100.00%	100.00%
Sphincter management - bladder	89.69%	25.69%	81.33%	92.31%	80.00%	64.29%
Sphincter management - bowel	94.40%	19.47%	90.67%	96.15%	88.57%	85.71%
Use of toilet	92.53%	19.94%	81.33%	100.00%	77.14%	57.14%
*Respiration and sphincter management subscale score*	93.80%	14.37%	72.00%	88.46%	71.43%	42.86%
Mobility in bed	95.56%	17.61%	93.33%	100.00%	94.29%	78.57%
Transfers: bed-wheelchair	96.00%	15.94%	93.33%	96.15%	94.29%	85.71%
Transfers: wheelchair-toilet	92.00%	21.81%	86.67%	96.15%	88.57%	64.29%
Mobility indoors	91.67%	19.53%	81.33%	96.15%	80.00%	57.14%
Mobility for moderate distances	90.50%	21.54%	80.00%	96.15%	80.00%	50.00%
Mobility outdoors	83.67%	31.90%	73.33%	88.46%	74.29%	42.86%
Stair management	84.89%	28.10%	70.67%	96.15%	68.57%	28.57%
Transfers: wheelchair-car	88.67%	27.96%	84.00%	96.15%	82.86%	64.29%
Transfers: ground-wheelchair	88.00%	32.71%	88.00%	100.00%	85.71%	71.43%
*Mobility subscale score*	89.90%	20.16%	64.00%	88.46%	60.00%	28.57%

*Note:* Data are presented as normalized (to maximum possible score per each item and each subscale, and to maximum possible total score) means and standard deviation, along with the percentage of assessments with maximum possible score for each item and subscale and for total score.

**Figure 3.**
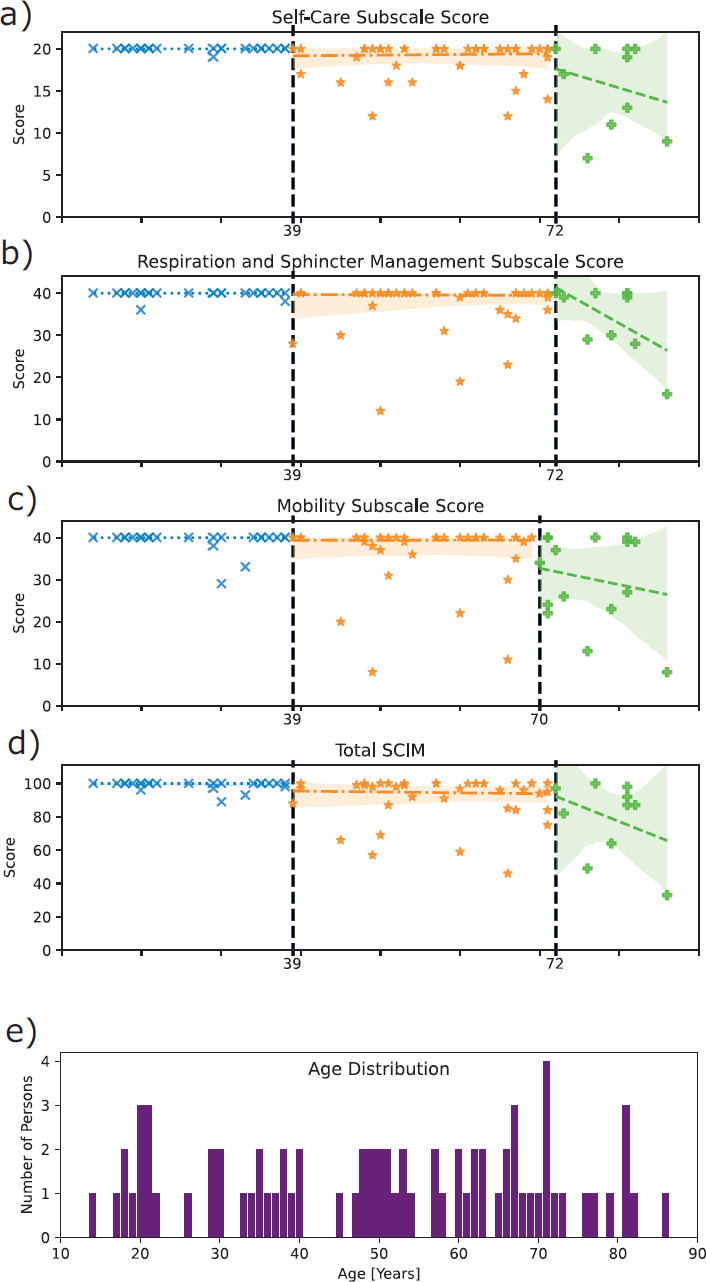
Age-aligned aggregate score distribution per Spinal Cord Independence Measure (SCIM) III subscale with detected change points in years, and age distribution of 75 individuals with SCIM III assessments at time of first American Spinal Injury Association Impairment Scale (AIS) E conversion. Predicted change points are marked with dashed lines. Robust linear regression was performed for the resulting intervals, marked in blue, orange, and green. Confidence intervals in lighter shades. a) Self-care interval slopes: b_C1_ = 4.02 * 10^-16^; b_C1-C2_ = 7.68 * 10^-3^; b_C2_ = −0.28. b) Respiration and sphincter management interval slopes: b_C1_ = −2.14 * 10^-16^; b_C1-C2_ = −6.59 * 10^-3^; b_C2_ = −1.07. c) Mobility interval slope: b_C1_ = −1.48 * 10^-17^; b_C1-C2_ = 1.38 * 10^-3^; b_C2_ = −0.39. d) Total SCIM intervals: b_C1_ = −9.86 * 10^-12^; b_C1-C2_ = −0.05; b_C2_ = −1.89. e) Age distribution in years of individuals classified as AIS E with SCIM III assessments at the first time of conversion.

The age range of the 75 individuals was 14 to 86 years (**Figure 3e**). Assuming 2 change points (equal to 3 age intervals), these were predicted at 39 and 72 years of age for self-care, respiration and sphincter management, and total SCIM and at 39 and 70 years for mobility.

Individuals were accordingly stratified into cohorts based on age. To account for the variation in the second predicted change point in mobility (**Figure 3c**) versus the rest (**Figure 3a, b, d**), the down-rounded arithmetic mean of all change points per segment was used. The resulting values of 39 and 71 years served as the boundaries for the following age cohorts: young (<40 years old; *n* = 26, age: 26.92 ± 7.95 years, 23.1% female, 92.3% traumatic), intermediate (40-70; *n* = 35, age: 56.80 ± 8.51 years, 17.1% female, 96.1% traumatic), and senior adults (>70; *n* = 14, age: 76.57 ± 5.12 years, 28.6% female, 85.7% traumatic), with no significant differences in sex and cause of SCI. Cohort boundaries and predicted change points are in strong agreement (self-care: Δ_HA_ = 4.0, Δ_RI_ = 0.91, F_mi_ = 0.93; respiration and sphincter management: Δ_HA_ = 4.0, Δ_RI_ = 0.91, F_mi_ = 0.93; mobility: Δ_HA_ = 1.0, Δ_RI_ = 0.96, F_mi_ = 1.0; total SCIM score: Δ_HA_ = 4.0, Δ_RI_ = 0.91, F_mi_ = 0.93).

Only 28.57% of individuals in the senior cohort achieved full independence in mobility compared to 88.46% of the young cohort (**Table 1**). The subscale with the closest proximity between the young and senior cohorts in full independence is respiration and sphincter management (45.60% difference; **Table 1**). For a breakdown of the statistics of every SCIM III item per cohort see eTable 2.

### Age cohort analysis

**Figure 4.**
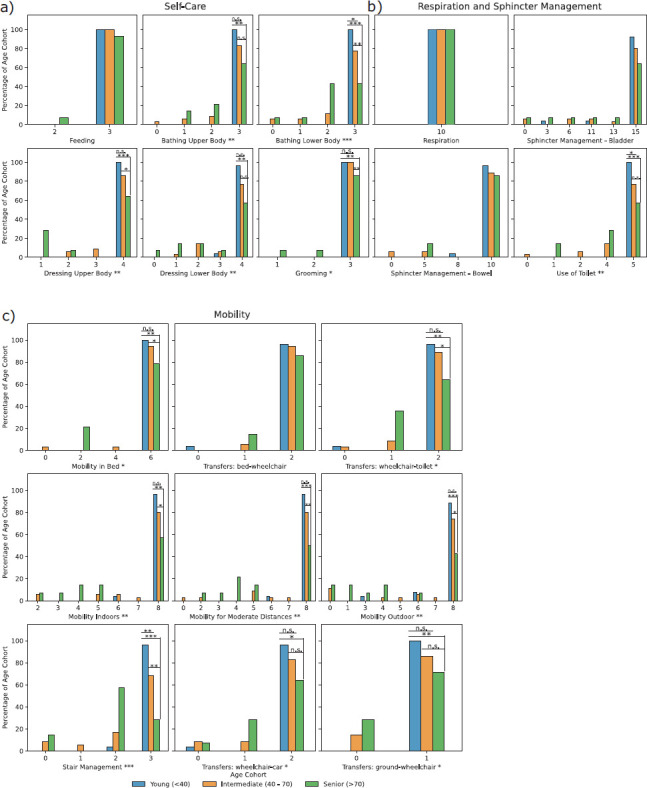
Histograms of the frequency of occurrence of Spinal Cord Independence (SCIM) III item grades for each of the three age cohorts (≤40, blue; >40 to <70, orange; ≥70, green). Kruskal-Wallis H-testing significance was marked (*P ≤ .05; **P ≤ .01; *** P≤ .001) in the SCIM item descriptor of a subplot if applicable. Post hoc pairwise Conover-Iman testing between the three age cohorts was performed in SCIM item grades and significance was marked above the bars of the highest score (*P ≤ .05; **P ≤ .01; *** P≤ .001). a) Five out of six self-care subscale items showed statistical significance: bathing upper body, bathing lower body, dressing upper body, dressing lower body, and grooming. b) Use of toilet was the only item with statistically significant differences out of four in respiration and sphincter management subscale. c) Eight out of 9 items of the mobility subscale featured statistical significance between age cohorts: mobility in bed; transfers: wheelchair-toilet; mobility indoors; mobility for moderate distances; mobility outdoors; stair management; transfers: wheelchair-car; and transfers: ground-wheelchair.

Differences between age cohorts in frequencies of grade occurrences were analyzed for each SCIM III item. Significant differences between age cohorts were detected across all categories in several items, such as dressing upper body (young, 4.00 ± 0.00; intermediate, 3.80 ± 0.53; senior, 3.00 ± 1.41; Kruskal-Wallis *P* ≤ .01), use of toilet (young, 5.00 ± 0.00; intermediate, 4.54 ± 1.09; senior, 4.14 ± 1.41; *P* ≤ .01), and stair management (young, 2.96 ± 0.20; intermediate, 2.46 ± 0.95; senior, 2.00 ± 0.96; *P* ≤ .001) (see eTable 2). Post hoc pairwise testing between cohorts was performed in items with significant differences (**Figure 4**).

The same analysis was performed across all SCIM subscale scores and total SCIM scores also revealing significant differences between age cohorts (see eFigure 1). Twenty of 75 individuals had a SCIM III assessment available after their initial AIS E conversion. An improvement in total SCIM and subscale scores was observed in the latest available SCIM III assessments versus the SCIM assessments at the time of initial AIS E conversion (initial AIS E total SCIM score: 89.40 ± 18.61, latest, 96.05 ± 15.27, Wilcoxon *P* ≤ .05; initial AIS E self-care score: 18.85 ± 3.03, latest: 19.30 ± 3.13, *P* > .05; initial AIS E respiration and sphincter management score: 36.15 ± 8.32, latest: 38.60 ± 5.39, *P* > .05; initial AIS E mobility score: 34.40 ± 10.35, latest: 38.15 ± 6.81, *P* ≤ .05).

## Discussion

AIS E is the rarest AIS grade in individuals with traumatic or ischemic SCI at 2% of all individuals in the EMSCI database, reflecting observations elsewhere.^16^-^18^ Conversion to AIS E occurred only in initially incomplete lesions (AIS B-D) approximately 6 months post injury and was directly preceded by mean total sensory and motor scores reaching 92.7% of their maximum. Analysis of the SCIM III assessments demonstrated that, while no normalized mean SCIM item score was lower than 83%, only approximately half (49.3%) of the 75 investigated individuals were scored with full functional independence at the point in time they were classified as AIS E.

Based on detected change points in age of SCIM III subscale and total scores, individuals in the dataset were stratified into 3 age cohorts (young, <40; intermediate, 40 to 70; senior, >70 years old). Stratification into cohorts revealed notably worse attainment of functional independence in all SCIM III items among the individuals of the senior cohort. Full functional independence in the mobility subscale was reached in only 28.6% of the individuals in the senior cohort compared to 60% in the intermediate and 88.5% in the young cohorts. Significant differences were found between those cohorts especially in the SCIM items of the mobility subscale, followed by the self-care and respiration and sphincter management subscales. Descriptive intraindividual comparison of the latest available SCIM III assessment and the one at the first AIS E conversion suggests ongoing improvement in functional independence, which is not reflected in the ISNCSCI exam having reached its ceiling.

The 2019 revision of ISNCSCI defines AIS E as “normal,” “if sensation and motor function as tested with the ISNCSCI are graded as normal in all segments.”^1^ However, the ISNCSCI does not assess other consequences of SCI, such as pain, spasticity, or impairments in proprioception,^19^ nor does grading of sensorimotor functions by the AIS reflect functional independence in everyday life tasks. The AIS was initially modified from the Frankel Scale representing a combined neurological and functional grading of SCI.^1^ Frankel grade E implies “that the patient was free of neurological *symptoms*, i.e. no weakness, no sensory loss, no sphincter disturbance,” but adds that “abnormal reflexes may have been present.”^20^ Based on our analysis, the principle implied in the definition of Frankel E grade that various functional and even sensorimotor symptoms, for example, weakness of hip abductor or adductor muscles, not tested in ISNCSCI may still be present is worth considering.

Our analysis shows that age plays a significant role in AIS E individuals’ restrictions to perform activities of daily living, with SCIM subscale and total SCIM scores having fair^15^ negative correlation to age. The idea that age has relevance in functional recovery of SCI individuals is not new: previous research by Naka et al.^21^ found significant differences in the SCIM III items mobility indoors and mobility outdoors in individuals classified as AIS D between the subgroup with age ≥70 years and the group with age <70 years.

Mobility issues are a matter of concern not only in people with motor incomplete SCI but also in the general aging population. A marked prevalence of self-reported mobility limitations in older people has been observed: for example, up to half of individuals aged 65 and over report problems in stairs-related mobility.^22^ A comprehensive literature review by Grimmer et al.^23^ showed, among other affected variables, a 21% decrease in level walking speed between ages 20 and 85, with the brunt of this decline (18%) occurring between 60 and 85 years. As such, our finding that older adults graded AIS E exhibit worse functional impairments than younger individuals fits into these observations from the general population.

More striking is the observation that offline change point prediction using SCIM III data predicts remarkably consistent ages: 39 years old as the first change point, and a range of 70-72 years old as the second. However, as many algorithms and cost functions exist for change point detection,^9^ the way the task has been implemented here should be considered explorative. The small size of the final dataset lent itself to using an algorithm suited for optimal rather than approximate determination of change points. A least squares deviation cost function is simple to interpret, but it may be sensitive to outliers, thus potentially introducing false positives. Additionally, the bimodal age distribution of the EMSCI cohort^10^ with peaks at the age of 28 and 60 might have an influence on the change point detection.

The small size of the finally analyzed ISNCSCI and SCIM dataset from individuals with at least one AIS E classification presents a significant limitation of this work. Regression, even using a method robust against outliers, and the values sourced from it should be considered cautiously: the slight trend for functional improvement in the intermediate cohort of SCIM self-care (**Figure 3a**, positive slope b_C1-C2_) and mobility (**Figure 3c**, positive slope b_C1-C2_) subscale scores is counterintuitive and may be an effect of the small sample size. Meanwhile, the plateau in the younger age interval and the negative slopes in the oldest interval suggest the concept nonetheless has veracity. As such, the regression should be considered demonstrative of a change in statistics within the segments rather than a means for prognostics. Similarly, the results of significance tests should be interpreted cautiously, and nonsignificant results should not be ignored.

A further limitation is that neither comorbidities, preinjury ambulation ability, nor the exact cause of injury were documented in the EMSCI database. It is known that secondary conditions, including fatigue, sarcopenia, osteoarthritis, cardiovascular diseases, or cognitive decline, occur more often in older than younger individuals without and with SCI,^24^,^25^ which could be an explanation for the drop in functional independence observed in the senior cohort. It is highly recommended to assess this complementary information in SCI registries for better prediction of functional outcomes.

## Conclusion

This work demonstrates that the current definition of AIS grade E insufficiently reflects the reality experienced by some people classified as AIS E. According to our observations, specifying AIS E not simply as “normal”^1^ but rather as “normal according to ISNCSCI” and clarifying that functional and neurological problems may persist better reflect this reality. This clarification further stresses that AIS E individuals should not be excluded in registry studies such as EMSCI and that assessments such as SCIM III or the International Standards to document Autonomic Function following SCI (ISAFSCI)^26^ are essential to determine whether any kinds of impairments on a body function and structure level remain. Age, particularly above 70 years, appears to be an important factor in the degree of functional impairment experienced. To detect whether functional deficits in the older subgroup are related to comorbidities attributable to aging rather than remnant deficits of SCI, functional assessment with SCIM should be performed in an age-matched non-SCI control group along with documentation of individual comorbidities. The availability of age-adjusted SCIM reference scores would support the definition of achievable rehabilitative goals in older people and the targeted selection of rehabilitative interventions in the presence of comorbidities.

## Supplementary Material






