# Supplementary material for: American Spinal Injury Association Impairment Scale Grade E Conversion After Spinal Cord Injury: Incidence, Conversion Characteristics, and Impact of Age on Functional Independence
Source: Top Spinal Cord Inj Rehabil. 2025 Aug 22;31(3):48–60. doi: 10.46292/sci25-00009 (PMC12376143; doi:10.46292/sci25-00009)
Supplement: Supplementary file 1 [file i1945-5763-31-3-48_s01.pdf]

eAPPENDIX A

Further Details on the European Multicenter Study about Spinal Cord Injury (EMSCI)

Governance, study design

Founded in 2001 with the coordinating centers of University Clinic Balgrist (Zurich, Switzerland) and Heidelberg University Hospital (Heidelberg, Germany), the EMSCI network comprises up to 26 participating trauma and spinal cord injury (SCI) rehabilitation centers from across Europe (Austria, Czech Republic, France, Germany, United Kingdom, Italy, Netherlands, Spain, Switzerland) and India (https://www.emsci.org/index.php/members). Collectively, the EMSCI network has collected data from more than 6,000 (end of 2024) individuals with traumatic or ischemic SCI. The EMSCI is an ongoing (registered in clinicaltrails.gov, NCT01571531) longitudinal, observational study that prospectively collects clinical, functional, and neurophysiological data with a standardized visit schedule over the first year of injury: very acute (within 2 weeks), acute I (1 month), acute II (3 months), and acute III (6 months), and chronic (12 months).

Inclusion and exclusion criteria

Three inclusion criteria have to be met before a patient can be enrolled in the EMSCI: (1) the patient has to be capable and willing to give written informed consent; (2) the spinal cord injury was caused by a single traumatic or ischemic event; and (3) the first EMSCI assessment was done within the first 6 weeks following injury.

Patients are excluded from EMSCI for the following reasons: (1) non-traumatic spinal cord injury (e.g., disc herniation, tumor, AV-malformation, myelitis) excl. single event ischemic incidences; (2) previously known dementia or severe reduction of cognitive functions, leading to reduced capabilities of cooperation or giving consent; (3) peripheral nerve injuries (e.g., plexus brachialis lesion) above the level of the SCI; (4) pre-existing polyneuropathy; and (5) severe craniocerebral injury. All individuals in the EMSCI receive standards of care.

Quality management

Since August 2010, EMSCI has been ISO 9001:2015-certified. To maintain a high level of data quality, the EMSCI network has implemented regular ISNCSCI instruction courses to their members. The training is instructed by experienced ISNCSCI examiners and raters. Analyses of standardized pre-course and post-course tests show that ISNCSCI trainings significantly improve classification skills (Schuld C, Wiese J, Franz S, et al. Effect of formal training in scaling, scoring and classification of the International Standards for Neurological Classification of Spinal Cord Injury. *Spinal Cord*.

2013 Apr;51(4):282-8. doi: 10.1038/sc.2012.149) and knowledge of the examination guidelines (Franz S, Heutehaus L, Weinand S, et al. Theoretical and practical training improves knowledge of the examination guidelines of the International Standards for Neurological Classification of Spinal Cord Injury. *Spinal Cord*. 2022;60(1):1-10. doi: 10.1038/s41393-020-00578-1) regardless of the experience in spinal cord injury medicine.

This supplementary digital material accompanies
Top Spinal Cord Inj Rehabil 2025;31(3):48-60
© 2025 American Spinal Injury Association
www.asia-spinalinjury.org
doi: 10.46292/sci25-00009
