# Supplementary material for: American Spinal Injury Association Impairment Scale Grade E Conversion After Spinal Cord Injury: Incidence, Conversion Characteristics, and Impact of Age on Functional Independence
Source: Top Spinal Cord Inj Rehabil. 2025 Aug 22;31(3):48–60. doi: 10.46292/sci25-00009 (PMC12376143; doi:10.46292/sci25-00009)
Supplement: Supplementary file 2 [file i1945-5763-31-3-48_s02.pdf]

eTable 1. All EMSCI American Spinal Injury Association Impairment Scale (AIS) grade E individual ($n = 82$) counts binned into year segments ranging from 2008-2012, 2013-2017, and 2018-2021

		Year segments		
		**2008-2012** (*n*=16)	**2013-2017** (*n*=31)	**2018-2021** (*n*=35)
**Exam stage of first AIS E classification**	**Very acute**	1 (6.25%)	0 (0.00%)	2 (5.71%)
	**Acute I**	6 (37.50%)	3 (9.68%)	3 (8.57%)
	**Acute II**	4 (25.00%)	8 (25.81%)	7 (20.00%)
	**Acute III**	2 (12.50%)	10 (32.26%)	7 (20.00%)
	**Chronic**	3 (18.75%)	10 (32.26%)	16 (45.71%)
**First AIS grade recorded**	**E**	5 (31.25%)	1 (3.23%)	6 (17.14%)
	**D**	10 (62.50%)	26 (83.87%)	26 (74.29%)
	**C**	0	2 (6.45%)	3 (8.57%)
	**B**	1 (6.25%)	2 (6.45%)	0
	**A**	0	0	0
**Sex**	**Female**	1 (6.25%)	8 (25.81%)	8 (22.86%)
	**Male**	15 (93.75%)	23 (74.19%)	27 (77.14%)
**Cause**	**Traumatic**	15 (93.75%)	28 (90.32%)	34 (97.14%)
	**Ischemic**	1 (6.25%)	3 (9.68%)	1 (2.86%)
**Age,** years		54.12±19.39	46.90±19.30	49.37±20.38

Note: Exam stage at first AIS E classification, first AIS recorded, sex and cause of spinal cord injury (SCI) are shown. Time frames of exam stages: very acute (0-15 days post injury), acute I (16-40 days), acute II (70-98 days), acute III (150-186 days), and chronic (300-546 days).

This supplementary digital material accompanies
Top Spinal Cord Inj Rehabil 2025;31(3):48-60
© 2025 American Spinal Injury Association
www.asia-spinalinjury.org
doi: 10.46292/sci25-00009

eTable 2. All Spinal Cord Independence (SCIM) III items per subscale (aggregate subscale scores and total SCIM score in italics) and age cohorts, with the number of individuals (total 75) per cohort stated

**SCIM III subscale**	**SCIM III item** (Score range)	**Young, <40 years** (n = 26)	**Intermediate, 40-70 years** (n = 35)	**Senior, >70 years** (n = 14)
	Total SCIM score (0-100)	98.50±3.57<br>100.0 (0.00)	91.69±14.38<br>99.0 (8.50)	81.64±20.22<br>89.5 (19.25)
Self-Care	Feeding (0-3)	3.00±0.00<br>3.0 (0.00)	3.00±0.00<br>3.0 (0.00)	2.93±0.27<br>3.0 (0.00)
	Bathing upper body (0-3)	3.00±0.00<br>3.0 (0.00)	2.71±0.71<br>3.0 (0.00)	2.50±0.76<br>3.0 (1.00)
	Bathing lower body (0-3)	3.00±0.00<br>3.0 (0.00)	2.60±0.85<br>3.0 (0.00)	2.21±0.89<br>2.0 (1.00)
	Dressing upper body (0-4)	4.00±0.00<br>4.0 (0.00)	3.80±0.53<br>4.0 (0.00)	3.00±1.41<br>4.0 (2.75)
	Dressing lower body (0-4)	3.96±0.20<br>4.0 (0.00)	3.57±0.85<br>4.0 (0.25)	2.93±1.44<br>4.0 (2.00)
	Grooming (0-3)	3.00±0.00<br>3.0 (0.00)	3.00±0.00<br>3.0 (0.00)	2.79±0.58<br>3.0 (0.00)
	Self-Care subscale score (0-20)	19.96±0.20<br>20.0 (0.00)	18.69±2.25<br>20.0 (2.00)	16.36±4.65<br>19.0 (6.75)
Respiration and Sphincter Management	Respiration (0-10)	10.00±0.00<br>10.0 (0.00)	10.00±0.00<br>10.0 (0.00)	10.00±0.00<br>10.0 (0.00)
	Sphincter management-bladder (0-15)	14.38±2.45<br>15.0 (0.00)	13.34±4.03,<br>15.0 (0.00)	12.00±5.14<br>15.0 (3.50)
	Sphincter management-bowel (0-10)	9.92±0.39<br>10.0 (0.00)	9.14±2.57<br>10.0 (0.00)	9.29±1.82<br>10.0 (0.00)
	Use of toilet (0-5)	5.00±0.00<br>5.0 (0.00)	4.54±1.09<br>5.0 (0.25)	4.14±1.41<br>5.0 (1.00)
	Respiration and Sphincter Management subscale score (0-40)	39.31±2.46<br>40.0 (0.00)	37.03±6.58<br>40.0 (2.00)	35.43±7.17<br>39.0 (8.50)

(continues)

eTable 2. All Spinal Cord Independence (SCIM) III items per subscale (aggregate subscale scores and total SCIM score in italics) and age cohorts, with the number of individuals (total 75) per cohort stated *(cont.)*

Mobility	Mobility in bed<br>(0-6)	6.00±0.00<br>6.0 (0.00)	5.77±1.06<br>6.0 (0.00)	5.14±1.70<br>6.0 (0.00)
	Transfers: bed-wheelchair	1.92±0.39	1.94±0.24	1.86±0.36
	(0-2)	2.0 (0.00)	2.0 (0.00)	2.0 (0.00)
	Transfers: wheelchair-toilet<br>(0-2)	1.92±0.39<br>2.0 (0.00)	1.86±0.43<br>2.0 (0.00)	1.64±0.50<br>2.0 (1.00)
	Mobility indoors<br>(0-8)	7.92±0.39<br>8.0 (0.00)	7.34±1.57<br>8.0 (0.00)	6.21±2.26<br>8.0 (3.75)
	Mobility for moderate distances<br>(0-8)	7.92±0.39<br>8.0 (0.00)	7.26±1.82<br>8.0 (0.00)	5.93±2.27<br>6.5 (4.00)
	Mobility outdoors<br>(0-8)	7.65±1.09<br>8.0 (0.00)	6.74±2.63<br>8.0 (0.50)	4.79±3.31<br>5.0 (6.50)
	Stair management<br>(0-3)	2.96±0.20<br>3.0 (0.00)	2.46±0.95<br>3.0 (1.00)	2.00±0.96<br>2.0 (0.75)
	Transfers: wheelchair-car<br>(0-2)	1.92±0.39<br>2.0 (0.00)	1.74±0.61<br>2.0 (0.00)	1.57±0.65<br>2.0 (1.00)
	Transfers: ground-wheelchair<br>(0-1)	1.00±0.00<br>1.0 (0.00)	0.86±0.36<br>1.0 (0.00)	0.71±0.47<br>1.0 (0.75)
	Mobility subscale score<br>(0-40)	39.23±2.52<br>40.0 (0.00)	35.97±8.22<br>40.0 (3.50)	29.86±10.95<br>32.0 (16.50)

Note: The cohorts are quantitatively described according to mean ± standard deviation, median and interquartile range (IQR) from 25% to 75%.
