# Supplementary material for: American Spinal Injury Association Impairment Scale Grade E Conversion After Spinal Cord Injury: Incidence, Conversion Characteristics, and Impact of Age on Functional Independence
Source: Top Spinal Cord Inj Rehabil. 2025 Aug 22;31(3):48–60. doi: 10.46292/sci25-00009 (PMC12376143; doi:10.46292/sci25-00009)
Supplement: Supplementary file 3 [file i1945-5763-31-3-48_s03.pdf]

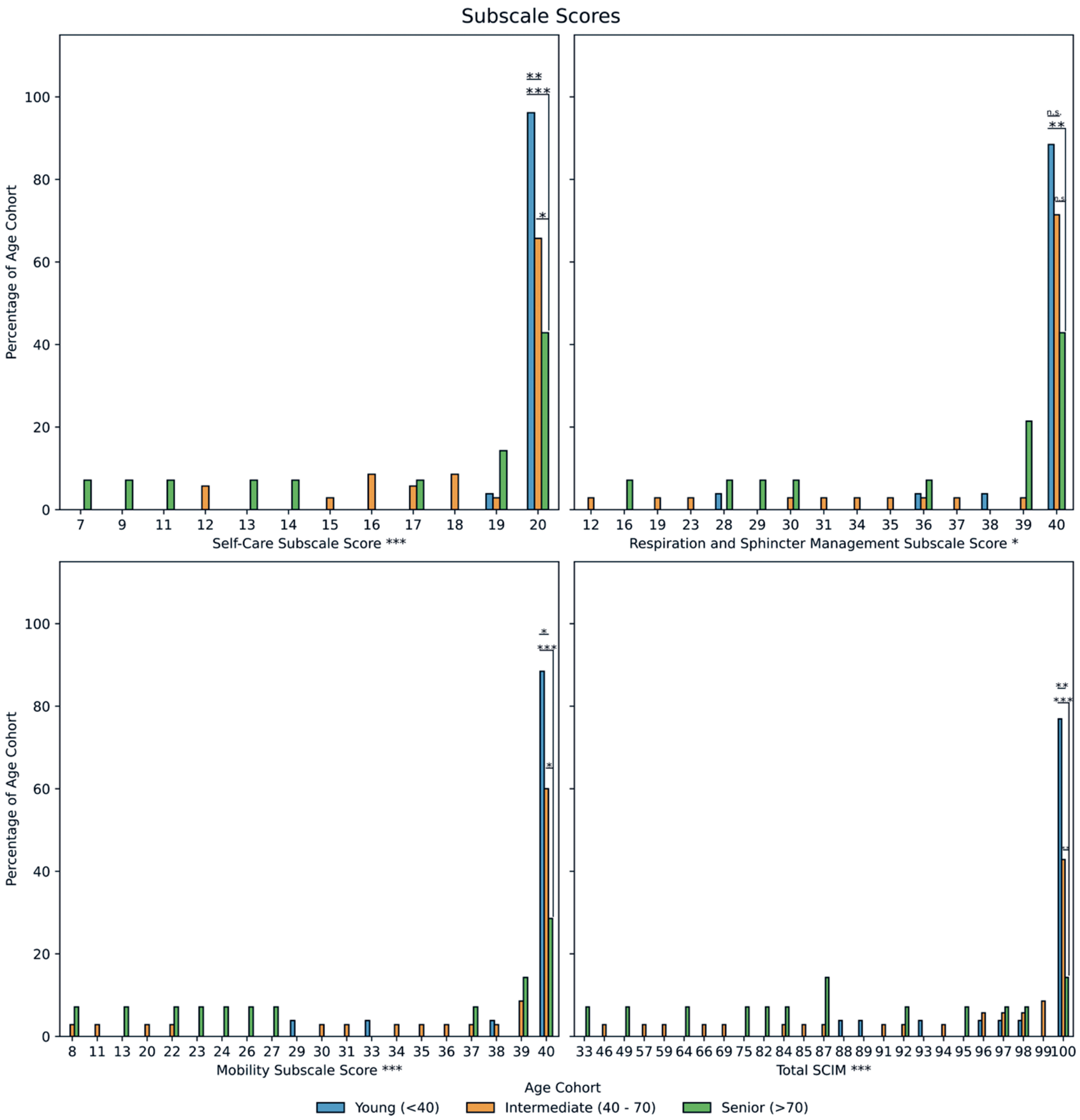

eFigure 1. Histograms of the frequency of occurrence of Spinal Cord Independence (SCIM) III subscale scores and total scores for each of the three age cohorts (Young, blue; Intermediate, orange; Senior, green). Significance (Kruskal-Wallis H-test) was marked (*$P \leq .05$; **$P \leq .01$; ***$P \leq .001$) in each of the SCIM subscale descriptors and the total score descriptor. Post hoc pairwise Conover testing between the three age cohorts was performed in SCIM subscale and total SCIM scores and significance marked above the bars of the highest score (*$P \leq .05$; **$P \leq .01$; ***$P \leq .001$). Significant differences were found in every SCIM subscale and total SCIM score.

This supplementary digital material accompanies
Top Spinal Cord Inj Rehabil 2025;31(3):48-60
© 2025 American Spinal Injury Association
www.asia-spinalinjury.org
doi: 10.46292/sci25-00009
